# Editorial: Building public confidence in innovative mRNA vaccines

**DOI:** 10.3389/fpubh.2025.1557596

**Published:** 2025-01-28

**Authors:** Jia Hu, Kenneth Rabin, Cora Constantinescu, Heidi J. Larson, Scott Ratzan

**Affiliations:** ^1^Community Health Sciences, University of Calgary, Calgary, AB, Canada; ^2^Graduate School of Public Health and Health Policy, The City University of New York, New York, NY, United States; ^3^Pediatric Infectious Diseases, Department of Pediatrics, Cumming School of Medicine, University of Calgary, Calgary, AB, Canada; ^4^London School of Hygiene and Tropical Medicine, London, United Kingdom; ^5^Institute for Health Metrics and Evaluation, University of Washington, Seattle, WA, United States

**Keywords:** vaccine confidence, mRNA vaccines, vaccine hesitancy, vaccines, public trust and confidence

When we began work on this series some 2 years ago, we were acutely aware of a hardening minority of global public opposition to vaccination. We did not, however, imagine that over the intervening months, vaccination in general, and mRNA vaccines in particular, would escalate into a political wedge issue that threatens to undermine the foundational role of immunization in public health.

Building public confidence in vaccines in general, and mRNA vaccines in particular, is more important now than ever. The rapid development and launch of COVID-19 vaccines was estimated to have saved over 14.4 million lives within the 1^st^ year of their availability (1). Unfortunately, the pandemic also led to an unprecedented politicization of public health that significantly eroded confidence in vaccinations (2). Confidence in mRNA vaccines has taken the hardest hit. For example, several US states have actively tried to undermine access to mRNA vaccines for COVID-19 (3), a Japanese Nursing Ethics Association has recently questioned the safety of self-amplifying mRNA vaccines (4), and a high-level government report on pandemic response in Slovakia has suggested banning mRNA vaccines altogether in that country (5).

A global study analyzing over 740,000 tweets on X (formerly Twitter) about mRNA vaccines and therapeutics found that 69.5% expressed negative sentiment, while only 13.0% were positive (6). The Global Listening Project, a large-scale initiative dedicated to generating insights into key dimensions of societal preparedness to build social cohesion and prepare society for times of crisis, found that in 2023, only 66% of people would accept a newly approved mRNA vaccine (7). Additionally, despite many studies highlighting the safety and effectiveness of mRNA COVID-19 vaccines in children (8), vaccination uptake in this population has been very low across multiple jurisdictions (9, 10).

These challenges obscure mounting evidence that mRNA vaccines have already led to significant public health benefits and could accomplish a great deal more given the platform's potential for rapid adaptation to address novel pathogens, as well as emerging applications for preventing and treating non-infectious disease, specifically in oncology. Delivering on this promise will require better understanding and management of issues around mRNA vaccine hesitancy, and we hope this series of studies from a range of countries and populations will help accomplish this objective.

Trust (or lack thereof) in governments drives confidence in COVID-19 and mRNA-based vaccines. A study in the Democratic Republic of Congo found that publicly vaccinating the head of state increased acceptance, but only among those who trusted the head of state and who were aware it occurred (Collart et al.). A US study found that, despite eroded trust in federal and public health agencies, disadvantaged communities maintained reasonable trust in the municipal government for accurate COVID-19 vaccine information (Shiman et al.). A Canadian study highlighted the important role that community organizations can play in supporting vaccine confidence as trusted purveyors of information insulated from people's mistrust in government (Ashfield et al.).

Several studies in this series look at vaccine acceptance in specific populations. A study assessing vaccine acceptance among cancer patients in Jordan found that key drivers of vaccine acceptance included concern around COVID-19 infection and strong peer encouragement to be vaccinated (AlMasri et al.). Another study assessing vaccine preferences among pregnant and lactating women in Bangladesh and Kenya and found that non-mRNA vaccines were preferred due to safety concerns driven by media coverage (Schue et al.). A study of Canadian healthcare providers highlighted the importance of specialized communications training on having vaccine conversations with patients and found that virtual simulation games could increase confidence in this context (Doucette et al.). Finally, an intervention study in younger Canadian adults showed that short videos about COVID-19 based on messaging that focused on either altruism or individualism could increase willingness to be vaccinated (Batra et al.).

This series of studies highlights the importance of adopting constructive approaches to restoring public health confidence in vaccination, particularly mRNA vaccines. Efforts should prioritize grass-roots interactions within countries and targeted populations utilizing tailored messaging and communications strategies that address the specific concerns expressed by these communities. In an era where vaccine confidence faces multifaceted challenges, it is critical to spotlight research that advances vaccine advocacy and explores innovative approaches to building community influence in our collective efforts.
